# Synthesis of adsorbent from *Tamarix hispida* and modified by lanthanum metal for fluoride ions removal from wastewater: Adsorbent characteristics and real wastewater treatment data

**DOI:** 10.1016/j.dib.2017.07.010

**Published:** 2017-07-08

**Authors:** Nasim Habibi, Parham Rouhi, Bahman Ramavandi

**Affiliations:** a Department of Chemical Engineering, Bushehr Branch, Islamic Azad University, Bushehr, Iran; b Environmental Health Engineering Department, Faculty of Health and Nutrition, Bushehr University of Medical Sciences, Bushehr, Iran

**Keywords:** *Tamarix hispida*, Glass factory, Fluoride ions, Adsorbent, lanthanum metal

## Abstract

This data article describes a facile method for production of an adsorbent from *Tamarix hispida* wasted wood and modified by lanthanum metal for fluoride ions removal from wastewater. The main characteristics of the adsorbent consist of BET surface area, functional groups, and elemental analysis is presented. The data for attenuating the pollutants from a real wastewater treatment which was provided from a glass factory is also represented. More than 90% of fluoride content of the real wastewater was treated by the adsorbent. Generally, these data would be informative for extend research aim to industrial wastewater treatment and those who work in the wastewater treatment plants.

**Specifications Table**

**Table:** 

Subject area	*Chemical engineering*
More specific subject area	*Environmental technology*
Type of data	*Table and image*
How data was acquired	*The BET surface area (Micromeritics model TriStar II-3020), scanning electron microscopy (SEM, Sirion from FEI), Fourier transform infrared (FTIR, NICOLET 5700-FTIR), and energy dispersive spectroscopy (EDS, Horiba EX-250, Japan) was applied to analyze the characteristics of the adsorbent. The fluoride ion concentration was measured by an expandable ion analysis (Orion EA 940 ion meter).*
Data format	*Analyzed*
Experimental factors	– *Tamirex hispida biochar was prepared from T. hispida wood at 105 °C during 24 h.* – *The dried wood chips were amended by lanthanum metal by pyrolyzed method at 350 °C for 3.5 h.* – *The final product was used for fluoride removal from a glass factory wastewater.*
Experimental features	*Synthesis Tamarix hispida biochar and modified by lanthanum metal for fluoride removal*
Data source location	*Bushehr, Iran*
Data accessibility	*Data provided with the article*

**Value of the data**

• A facile method for modification of the biochar *Tamarix hispida* by lanthanum chloride is provided in this dataset compare to other biomass/adsorbents reported in the literature [1], [2], [3].
• The data may be useful for future researches that aim to glass factory wastewater treatment.
• This data allows wastewater treatment plants managers and engineers to extend the practical usage of the adsorbent.

## 1 Data

**Fig. 1:**
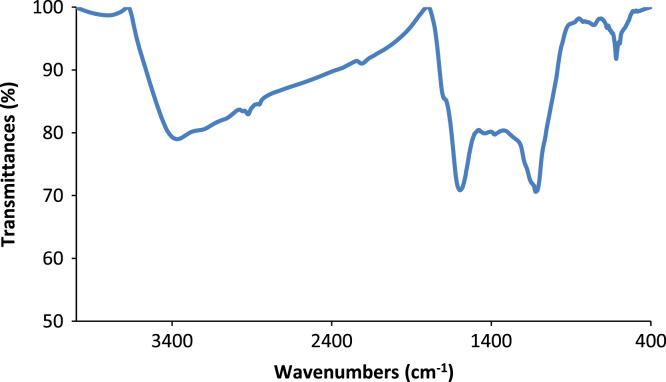
FTIR spectrum of the adsorbent from *Tamarix hispida* and modified by lanthanum chloride.

**Fig. 2:**
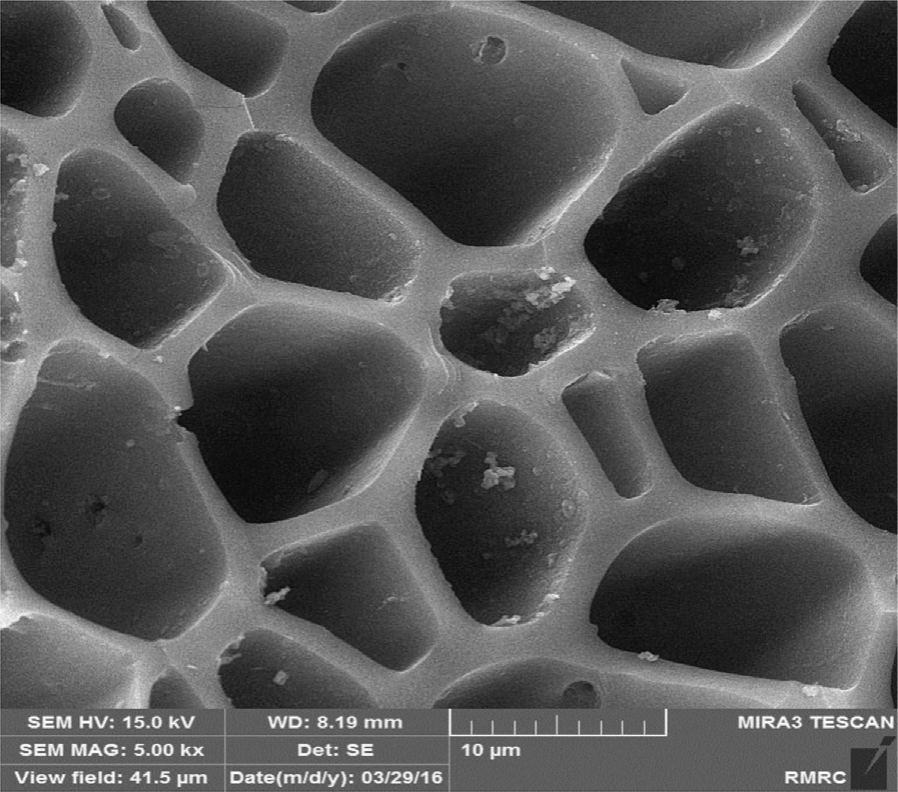
SEM image of the adsorbent from *Tamarix hispida* and modified by lanthanum metal.

**Fig. 3:**
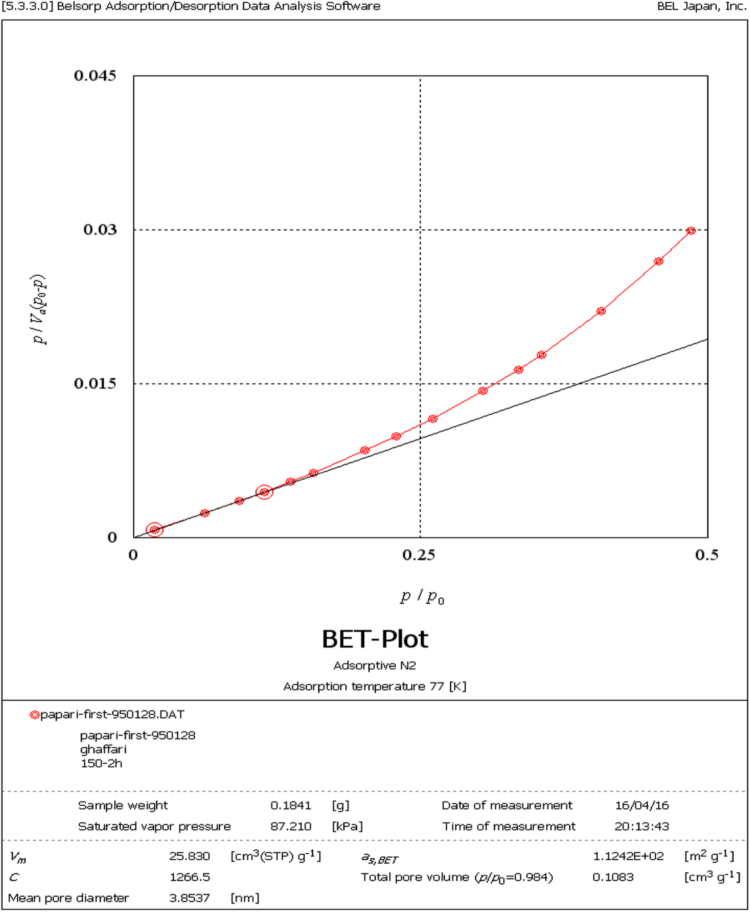
N_2_ adsorption–desorption isotherm of adsorbent prepared from *Tamarix hispida* and modified by lanthanum metal.

**Fig. 4:**
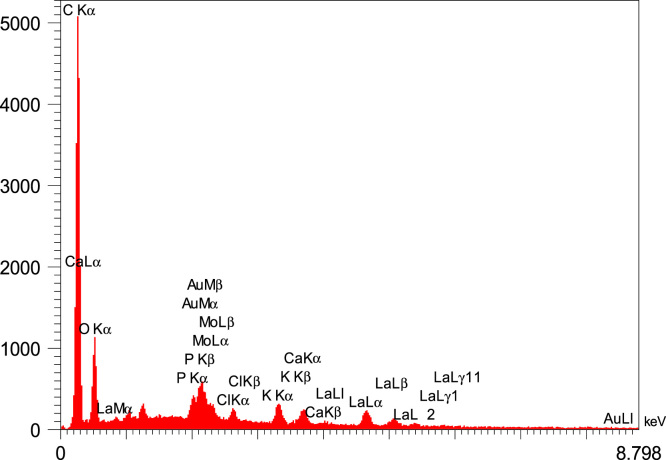
The EDS spectrum of the adsorbent from *Tamarix hispida* and modified by lanthanum metal.

The data regarding to potential of the adsorbent prepared from *T. hispida* and modified by lanthanum chloride for treating of the glass factory wastewater are presented in Table 1. The information about main characteristics of the adsorbent including FTIR spectrum, SEM image, N_2_ adsorption–desorption isotherm, and EDS spectrum are also shown in Fig. 1, Fig. 2, Fig. 3, Fig. 4.

**Table 1:** The quality of glass factory wastewater before and after treatment by the prepared adsorbent.

Wastewater quality	Concentration before treatment (mg/l)	Concentration after treatment (mg/l)
F^−^	23	2
NO_3_^−^	69	44
Cl^−^	212.5	54
SO_4_^−^	124	120
Total hardness	458	438
TDS	1060	905
BOD_5_	285	273.4
COD	564	555
pH	7.9	8.1

## 2 Experimental design, materials and methods

### 2.1 Adsorbent preparation

Dried *T. hispida* wood which was collected from surrounding area of Bushehr city was applied the initial material for the adsorbent providing. The method for production of the adsorbent from *T. hispida* was similar to methods provided in our previous studies [4], [5], [6]. The *T. hispida* wood was initially debarked and then chopped up into small pieces (1–3 cm). The wood chips were put in an oven (Shimaz Co., Iran) with 105 °C during 24 h. The dried chips were then milled and the achieved particles were amended by lanthanum chloride according to methods in the literature [7], [8], [9]. Briefly, 10 g of the particles produced in the previous stage and 0.27 g of lanthanum was poured in a 250 ml flasks and the amount of 150 ml double distilled water with pH of 12 was added to the flasks. The mixture was then agitated by a shaker incubator (Parsazma Co., Iran) at 150 rpm for 24 h. After this time, the solution was filtered by 0.42µ-Whatman filters. The particles trapped on the filter surface was dried at 105 °C during 24 h and then pyrolyzed at 350 °C for 3.5 h in the absence of oxygen. After that, the pyrolyzed pieces were milled and finally passed through 40 and 120 mesh ASTM sieves to obtain powder with diameters of 0.125–0.4 mm and used as adsorbent in the experiments.

### 2.2 Real wastewater sampling

A bulk of glass factory wastewater was provided from a factory around Shiraz city, Iran. This wastewater was treated by the prepared adsorbent. For this purpose, 150 ml wastewater and 10 g/l adsorbent was poured in 250 ml flasks and after a 60 min contact time the solutions were filtered. The original pH of the wastewater was changed during the experiment. The physic-chemical properties of the filtrate were analyzed (see Table 1). This test was repeated three times and average values were stated.

### 2.3 Analysis

The BET specific surface area the adsorbent was carried out by the N_2_ adsorption/desorption method at −196 °C using a Micromeritics model TriStar II-3020 instrument. For this purpose, the adsorbent samples were degassed for around 24 h at environment with temperature of 250 °C to remove any adsorbed contaminants or water content that might have been exist on the surface. Micrograph of pristine adsorbent was obtained by scanning electron microscopy (SEM, Sirion from FEI). For determination the functional groups on the adsorbent surface a Fourier transform infrared (FTIR) spectrometer (NICOLET 5700-FTIR) was applied. The elemental composition of the adsorbent provided from *T. hispida* and modified by lanthanum chloride was achieved by energy dispersive spectroscopy (EDS, Horiba EX-250, Japan). The fluoride concentration of the real wastewater sample was analyzed by an expandable ion analysis (Orion EA 940 ion meter) according to the method presented in the Standard Methods for the Examination of Water and Wastewater [10].

## References

[1] Saberzadeh Sarvestani F., Esmaeili H., Ramavandi B. (2016). Modification of Sargassum angustifolium by molybdate during a facile cultivation for high-rate phosphate removal from wastewater: structural characterization and adsorptive behavior. 3 Biotech.

[2] Hajivandi A., Farjadfard S., Ramavandi B., Akbarzadeh S. (2016). Experimental data for synthesis of bi-metalized chitosan particle for attenuating of an azo dye from wastewater. Data Brief.

[3] Xu L., Chen G., Peng C., Qiao H., Ke F., Hou R., Li D., Cai H., Wan X. (2017). Adsorptive removal of fluoride from drinking water using porous starch loaded with common metal ions. Carbohyd. Polym..

[4] Ramavandi B., Asgari G., Faradmal J., Sahebi S., Roshani B. (2014). Abatement of Cr (VI) from wastewater using a new adsorbent, cantaloupe peel: taguchi L16 orthogonal array optimization. Korean J. Chem. Eng..

[5] Rezaee A., Ramavandi B., Ganati F. (2006). Equilibrium and spectroscopic studies on biosorption of mercury by algae biomass. Pak. J. Biol. Sci..

[6] Khademi Z., Ramavandi B., Ghaneian M.T. (2015). The behaviors and characteristics of a mesoporous activated carbon prepared from *Tamarix hispida* for Zn(II) adsorption from wastewater. J. Environ. Chem. Eng..

[7] Asgari G., Ramavandi B., Farjadfard S. (2013). Abatement of azo dye from wastewater using bimetal-chitosan. Sci. World J..

[8] Mortazavi S.B., Ramavandi B., Moussavi G. (2011). Chemical reduction kinetics of nitrate in aqueous solution by Mg/Cu bimetallic particles. Environ. Technol..

[9] Asgari G., Ramavandi B., Sahebi S. (2014). Removal of a cationic dye from wastewater during purification by Phoenix dactylifera. Desalination Water Treat..

[10] Federation W.E., A.P.H. Association (2005). Standard Methods for the Examination of Water and Wastewater.

